# Predictors and Patterns of Metastatic *Candidozyma auris* Infection, Spain, 2016–2024

**DOI:** 10.3201/eid3211.260984

**Published:** 2026-11

**Authors:** Pedro Suarez-Urquiza, Alba Ruiz-Gaitán, Eva Calabuig, María Tasias, Víctor García Bustos, Ignacio Moreno, María Ángeles Ferre, Ana María Moret, Juan Frasquet, Patricio Favier, Javier Pemán, Maria Jesus Castaño-Aroca, Paula Muñoz Brell, Mónica Gordon, Maria Dolores Gomez-Ruiz, Miguel Salavert-Lleti

**Affiliations:** Hospital Universitari i Politècnic La Fe, Valencia, Spain (P. Suarez-Urquiza, A. Ruiz-Gaitán, E. Calabuig, M. Tasias, V. García Bustos, I. Moreno, M.Á. Ferre, A.M. Moret, J. Frasquet, P. Favier, J. Pemán, M.J. Castaño-Aroca, P. Muñoz Brell, M. Gordon, M.D. Gomez-Ruiz, M. Salavert-Lletí); Instituto de Investigación Sanitaria La Fe, Valencia (P. Suarez-Urquiza, A. Ruiz-Gaitán, E. Calabuig, M. Tasias, V. García Bustos, J. Frasquet, P. Favier, J. Pemán, P. Muñoz Brell, M. Gordon, M.D. Gomez-Ruiz, M. Salavert-Lletí); Universitat de València, Valencia (E. Calabuig)

**Keywords:** candidemia, Candida auris, Candidozyma auris, fungi, fungemia, endocarditis, antifungal agents, antimicrobial resistance, fungal, tertiary care centers, retrospective studies, risk factors, Spain

## Abstract

Metastatic infection after *Candidozyma auris* candidemia remains poorly characterized. We retrospectively studied patients with *C. auris* candidemia at a tertiary hospital in Spain during 2016–2024. Of 108 patients, 24 (22.2%) experienced metastatic infections; endocarditis was most frequent (7.4%), followed by osteoarticular infection, meningitis, and hepatosplenic infection (3.7% each). In multivariable Fine–Gray regression, a cardiac device or prosthetic valve was independently associated with metastatic infection (subdistribution hazard ratio [HR] 2.89 [95% CI 1.21–6.91]), although that association was attenuated in a sensitivity analysis restricted to echocardiographed patients. Persistent candidemia showed a higher, nonsignificant risk for metastatic infection in the day-5 landmark analysis (subdistribution HR 2.67 [95% CI 0.86–8.32]). Metastatic infection was associated with increased deaths in time-dependent analyses at 60 days (adjusted HR 2.21 [95% CI 1.04–4.67]) and 90 days (adjusted HR 2.26 [95% CI 1.09–4.67]). Our findings support echocardiography in patients with intracardiac devices.

Invasive candidiasis, in particular candidemia, is a significant infectious threat, especially among patients in intensive care units (ICUs). It represents between 15%–30% of infections in these units and, despite improvements in diagnosis and treatment, the mortality rate associated remain high, 30%–60% in most series (*1*,*2*). In that context, metastatic complication, in which infection spreads through the bloodstream to other organs, plays a key role. The most common manifestations include endophthalmitis, osteomyelitis, hepatosplenic abscesses, meningitis, and endocarditis (*3*). Depending on underlying clinical conditions and the screening protocols used, 4%–50% of candidemia cases develop metastatic involvement (*4*–*7*), which is frequently associated with increased risk for death and prolonged hospitalization (*8*).

*Candidozyma auris* (formerly *Candida auris*) (*9*) is an emerging, often multidrug-resistant yeast often associated with nosocomial outbreaks worldwide (*10*,*11*). It can colonize patients and healthcare environments. Since its identification in Japan in 2009 (*12*), *C. auris* has been reported on all continents except Antarctica and is often associated with high mortality rates. Most research to date has focused on its antifungal resistance patterns, outbreak dynamics, and infection control measures (*11*). However, its ability to cause metastatic complications, such as ocular involvement, endocarditis, osteomyelitis, or hepatosplenic candidiasis, is poorly characterized. Available evidence is largely confined to isolated case reports and small series, in which endocarditis in recipients of left ventricular assist devices and central nervous system infection associated with neurosurgical devices predominate (*13*–*23*). A few cohort studies have reported secondary organ involvement in 7.7%–27% of patients with *C. auris* candidemia, but they rarely specify the spectrum of complications, their predictors, or their outcomes (*18*–*20*,*22*). Further investigation is essential to clarify its clinical spectrum and the burden of secondary organ-specific infections. In this study, we aimed to characterize clinical and microbiological features of patients with metastatic *C. auris* infection after candidemia and to identify factors associated with metastatic involvement.

## Methods

### Study Design and Setting

We conducted this retrospective observational study at the Hospital Universitari i Politècnic La Fe, a 900-bed tertiary institution in Valencia, Spain, during May 2016–December 2024. *C. auris* was first detected in the institution in May 2016 during a hospital outbreak, and it has remained endemic since then, affecting both ICU and non-ICU patients (*24*).

### Patient Selection and Definitions

We identified all episodes of *Candidozyma auris* candidemia occurring in adult patients (>18 years of age) during the study period through the laboratory information system. We then manually reviewed the electronic medical records of the corresponding patients to collect demographic, clinical, microbiological, treatment, and outcome data.

We classified patients according to the presence or absence of metastatic infection, defined as compatible clinical manifestations together with either microbiological confirmation from a normally sterile site, radiologic findings consistent with metastatic infection, or histopathologic evidence. In addition, the presumed source of candidemia had to be unknown or anatomically distinct from the metastatic site. We defined persistent candidemia as the recovery of *C. auris* from >1 blood culture obtained on or after day 5 after the index positive blood culture. We considered the date of the index blood culture day 0.

### Microbiology

We identified *C. auris* isolates using classical microbiological phenotypic methods combined with matrix-assisted laser desorption/ionization-time of flight (MALDI-TOF) mass spectrometry (Bruker, https://www.bruker.com). We conducted antifungal susceptibility testing using the commercial microdilution method Sensitive YeastOne (Thermo Fisher Scientific, https://www.thermofisher.com) and interpreted results in accordance with the tentative breakpoints for *C. auris* established by the US Centers for Disease Control and Prevention (*25*), because the Clinical and Laboratory Standards Institute has not defined species-specific clinical breakpoints.

### Statistical Analysis

We compared patients with metastatic *C. auris* infection after candidemia to those with confirmed candidemia without evidence of dissemination, to assess clinical outcomes and potential associated factors. We analyzed categorical variables using χ^2^ test or Fisher exact test and continuous variables using unpaired *t*-test or Mann-Whitney-Wilcoxon test, as appropriate.

For the competing-risk analysis, follow-up began on the date of the index positive blood culture. The event of interest was the diagnosis of metastatic infection; we considered death before the diagnosis of metastatic infection a competing event. We followed patients who remained alive without documented metastatic infection through their last documented hospital contact. We estimated cumulative incidence functions accounting for death as a competing event. We developed a multivariable Fine–Gray regression model; we included variables with p<0.1 in bivariable analysis, together with variables considered clinically relevant, to estimate subdistribution hazard ratios (HRs) and 95% CI. To assess potential diagnostic bias related to echocardiography, we performed a sensitivity analysis restricted to patients who underwent echocardiographic evaluation. To address immortal time bias in the assessment of candidemia persistence, we performed a landmark analysis restricted to patients who survived beyond day 5 after the index blood culture. To further evaluate the association between metastatic infection and mortality, we performed a cause-specific Cox regression analysis for 30-day, 60-day, and 90-day all-cause mortality recorded for all patients from electronic and regional health records irrespective of hospital discharge, treating metastatic infection as a time-dependent exposure. Patients contributed person-time to the non–metastatic infection group until diagnosis and to the metastatic infection group thereafter. We included age and Charlson Comorbidity Index score in an adjusted model. We performed statistical analysis using R version 4.5.0 (The R Project for Statistical Computing, https://www.r-project.org); we considered p<0.05 statistically significant.

## Results

We included a total of 108 patients with *C. auris* candidemia. Among them, 24 (22.2%) experienced metastatic infection. Forty-four (40.7%) patients died without metastatic complications; we treated their deaths as competing events. Forty (37.0%) patients remained alive without documented metastatic infection; follow-up for those patients ended at their last available hospital discharge date. The median age was 60.6 (interquartile range [IQR] 48.9–72.0) years, and 78 (72.2%) were admitted to the ICU. 

No patient experienced >1 metastatic complication. The most frequent metastatic complication was endocarditis, observed in 8 (7.4%) patients, followed by osteoarticular infection (4 [3.7%]), meningitis (4 [3.7%]), and hepatosplenic infection (4 [3.7%]). Chorioretinitis was documented in 1 (0.9%) patient. Three (2.8%) additional patients experienced other late-onset complications such as aneurysm sac infection and subcutaneous abscess. Among the 24 patients with metastatic infections, the diagnosis was supported by imaging findings in 18 (75.0%) patients, microbiological confirmation from the metastatic site in 19 (79.2%) patients, and histopathologic evidence in 4 (16.7%) patients; those diagnostic modalities were not mutually exclusive. Most (84 [77.8%]) patients showed no evidence of metastatic involvement beyond the initial bloodstream infection.

### Risk Factors for Complications

In bivariable analysis, patients who had metastatic infections were older (median age 68.9 [IQR 54.4–75.5] vs. 59.7 [IQR 43.9–70.7] years; p = 0.065) and had a longer duration of candidemia (median 6 [IQR 1–38] vs. 1 [IQR 1–5] days; p = 0.034) than those without metastatic complications. A cardiac device or prosthetic valve was significantly more frequent in patients with metastatic infection than in those without (54.2% vs. 26.2%; p = 0.020). Among patients with cardiac devices or prosthetic valves, prosthetic heart valves were the most frequent subtype in both groups, those with metastatic infection and those without (6/13 [46.2%] vs. 12/22 [54.5%]), followed by permanent pacemakers or implantable cardioverter-defibrillators (5/13 [38.5%] vs. 10/22 [45.5%]). Left ventricular assist devices were present in 2/13 (15.4%) patients with metastatic infections and in none of the patients without. The distribution of device subtypes did not differ significantly between groups (p = 0.266). Echocardiography was performed significantly more frequently in patients with complications (87.5% vs. 63.1%; p = 0.026). The frequency of fundoscopic examination did not differ significantly between patients with and without metastatic infections (83.3% vs. 64.3%; p = 0.087).

We reviewed the initial management of candidemia. We found no significant differences in early central venous catheter removal (58.3% vs. 52.4%; p = 0.777), initiation of antifungal therapy within 48 hours (75.0% vs. 85.7%; p = 0.352), or resolution of the infectious focus within 48 hours (12.5% vs. 19%; p = 0.557). Initial antifungal treatment consisted predominantly of echinocandin monotherapy, administered to 62.5% of patients with and 78.6% of those without metastatic infections. Amphotericin B monotherapy was used in 8.3% of patients with and 1.2% of those without metastatic infections. No patients with metastatic infections received isavuconazole monotherapy; 1.2% of patients without metastatic infections received it. Initial combination antifungal therapy was administered to 29.2% of patients with metastatic infections and 19.0% of those without (p = 0.432). The most frequent combinations were an echinocandin plus isavuconazole (16.7% vs. 8.3%) and an echinocandin plus amphotericin B (12.5% vs. 10.7%). We observed no statistically significant differences in initial antifungal therapy between groups or in patients with concurrent or underlying conditions or baseline risk factors (Table 1).

**Table 1:** Baseline characteristics of patients with *Candidozyma auris* candidemia in study of metastatic *C. auris* infection, Spain, 2016–2024*

Variable	Total, n = 108	Metastatic infection absent, n = 84	Metastatic infection present, n = 24	p value
Median age, y (IQR)	60.6 (48.9-72.0)	59.7 (43.9–70.7)	68.9 (54.4–75.5)	0.065
Charlson Comorbidity Index, median (IQR)	5 (3–7.2)	5 (2.8–7.2)	5.5 (3–7.2)	0.465
Sex				0.757
M	76 (70.4)	58 (69)	18 (75)	
F	32 (29.6)	26 (31)	6 (25)	
Surgery in the past 30 d	44 (40.7)	30 (35.7)	14 (58.3)	0.080
Previous therapy				
Broad-spectrum antibiotics	100 (92.6)	80 (95.2)	20 (83.3)	0.071
Previous antifungal treatment	30 (27.8)	25 (29.8)	5 (20.8)	0.547
Corticosteroid therapy	21 (19.4)	14 (16.7)	7 (29.2)	0.284
Other immunosuppressive therapy	18 (16.7)	14 (16.7)	4 (16.7)	Referent
Risk factors				
Persistent candidemia	34 (31.5)	23 (27.4)	11 (45.8)	0.142
Median duration of candidemia, d (IQR)	1 (1–8)	1 (1–5)	6 (1–38)	0.034
Central venous catheter	93 (86.1)	72 (85.7)	21 (87.5)	Referent
Previous multisite colonization	81 (75)	64 (76.2)	17 (70.8)	0.789
Parenteral nutrition	68 (63)	53 (63.1)	15 (62.5)	Referent
Invasive mechanical ventilation	52 (48.1)	37 (44)	15 (62.5)	0.173
Cardiac device/prosthetic valve	35 (32.4)	22 (26.2)	13 (54.2)	0.020
Prosthetic heart valve	18 (51.4)	12 (54.5)	6 (46.2)	
PPM/ICD	15 (42.9)	10 (45.5)	5 (38.5)	
Left ventricular assist device	2 (5.7)	0 (0)	2 (15.4)	
Solid organ transplant	6 (5.6)	3 (3.6)	3 (12.5)	0.122
Severe sepsis	24 (22.2)	19 (22.6)	5 (20.8)	Referent
Osteoarticular prosthesis	15 (13.9)	11 (13.1)	4 (16.7)	0.739
Hemodialysis	15 (13.9)	11 (13.1)	4 (16.7)	0.739
Previous candidemia	10 (9.3)	6 (7.1)	4 (16.7)	0.224
Profound neutropenia, <500 cells/µL	7 (6.5)	4 (4.8)	3 (12.5)	0.182
Concomitant bacteremia	6 (5.6)	6 (7.1)	0 (0)	Referent
Admission department				
Surgical intensive care unit	57 (52.8)	47 (56)	10 (41.7)	0.460
Medical intensive care unit	21 (19.4)	15 (17.9)	6 (25)	
Other	30 (27.8)	22 (26.2)	8 (33.3)	
Management approach to candidemia				
Catheter removed within 48 h	58 (53.7)	44 (52.4)	14 (58.3)	0.777
Non-catheter focus resolution within 48 h	19 (17.6)	16 (19)	3 (12.5)	0.557
Cardiac echocardiography performed	74 (68.5)	53 (63.1)	21 (87.5)	0.026
Fundoscopic examination performed	74 (68.5)	54 (64.3)	20 (83.3)	0.087
Initial antifungal therapy				
Antifungal therapy within 48 h	90 (83.3)	72 (85.7)	18 (75)	0.352
Echinocandin monotherapy	81 (75)	66 (78.6)	15 (62.5)	0.181
Amphotericin B monotherapy	3 (2.8)	1 (1.2)	2 (8.3)	0.123
Isavuconazole monotherapy	1 (0.9)	1 (1.2)	0 (0)	Referent
Initial dual antifungal therapy	23 (21.3)	16 (19)	7 (29.2)	0.432
Echinocandin + isavuconazole	11 (10.2)	7 (8.3)	4 (16.7)	0.258
Echinocandin + amphotericin B	12 (11.1)	9 (10.7)	3 (12.5)	0.727
Outcome				
Median duration of antifungal therapy, d (IQR)	26.5 (15–35.2)	23 (15.0–32.2)	43 (4.0–72.2)	<0.001
Median length of hospital stay after candidemia, d (IQR)	23 (15.5–42.5)	22.5 (15.2–29.8)	47 (16.0–66.0)	0.052
Deaths within 30 d	37 (34.3)	32 (38.1)	5 (20.8)	0.184
Deaths within 60 d	44 (40.7)	36 (42.9)	8 (33.3)	0.547
Deaths within 90 d	50 (46.3)	40 (47.6)	10 (41.7)	0.777

*Values are no. (%) except as indicated. ICD, implantable cardioverter-defibrillators; IQR, interquartile range Q1–Q3; PPM, permanent pacemakers.

We performed multivariable Fine–Gray competing risks analysis with death as a competing event; the presence of a cardiac device or prosthetic valve was the only factor independently associated with the development of metastatic complications (subdistribution HR [sHR] 2.89 [95% CI 1.21–6.91]; p = 0.017). Age, previous candidemia, and recent surgery were not independently associated with metastatic infection (Table 2). In a sensitivity analysis restricted to patients who underwent echocardiography, the presence of a cardiac device or prosthetic valve was associated with metastatic infection in the unadjusted Fine–Gray model (sHR 2.32 [95% CI 1.01–5.33]; p = 0.046). After adjustment for age, previous candidemia, and recent surgery, the estimated association remained elevated but was not statistically significant (sHR 2.15 [95% CI 0.90–5.15]; p = 0.085).

**Table 2:** Multivariable Fine–Gray competing risks regression analysis of factors associated with metastatic infection in patients with *Candidozyma auris* candidemia, Spain, 2016–2024*

Variable	sHR (95% CI)	p value
Previous candidemia	1.61 (0.54–4.79)	0.412
Age, per year	1.02 (0.99–1.05)	0.241
Recent surgery	0.79 (0.33–1.90)	0.603
Cardiac device/prosthetic valve	2.89 (1.21–6.91)	0.017

*sHR, subdistribution hazard ratio.

In the landmark analysis performed including only patients alive at day 5 (n = 97), persistent candidemia at day 5 was associated with a higher risk for metastatic complications, although that association was not statistically significant (sHR 2.67 [95% CI 0.86–8.32]; p = 0.091). The presence of a cardiac device or prosthetic valve remained an independent predictor (sHR 4.04 [95% CI 1.19–13.7]; p = 0.025) (Table 3).

**Table 3:** Landmark analysis of factors associated with metastatic infection in patients with *Candidozyma auris* candidemia, Spain, 2016–2024*

Variable	sHR (95% CI)	p value
Persistent candidemia at day 5	2.67 (0.86–8.32)	0.091
Age, per year	1.02 (0.98–1.06)	0.413
Previous candidemia	1.37 (0.31–6.13)	0.688
Cardiac device/prosthetic valve	4.04 (1.19–13.7)	0.025
Recent surgery	0.99 (0.32–3.07)	0.943

*Analysis restricted to patients surviving beyond day 5 (n = 97). Death was retained as a competing event. sHR, subdistribution hazard ratio.

### Patient Outcomes

Crude mortality rate at 30 days after *C. auris* candidemia was 34.3%; at 60 days, 40.7%; and at 90 days, 46.3%. We found no significant differences between patients with and without metastatic complications; 30-day rates were 20.8% vs. 38.1% (p = 0.184); 60-day: 33.3% vs. 42.9% (p = 0.547); 90-day: 41.7% vs. 47.6% (p = 0.777). In cause-specific Cox regression analyses, metastatic infection, treated as a time-dependent exposure, was not significantly associated with 30-day mortality after adjustment for age and Charlson Comorbidity Index (adjusted HR [aHR] 1.75 [95% CI 0.58–5.29]; p = 0.323). However, metastatic infection was independently associated with higher risk for death at 60 days (aHR 2.21 [95% CI 1.04–4.67]; p = 0.038) and 90 days (aHR 2.26 [95% CI 1.09–4.67]; p = 0.028) (Table 4). In addition, patients with metastatic complications required significantly prolonged antifungal therapy (median 43 [IQR [4.0–72.2] days vs. 23 [IQR 15.0–32.2] days; p<0.001) and longer hospital stays, although the difference was not statistically significant (median 47 [IQR 16.0–66.0] days vs. 22.5 [IQR 15.2–29.8] days; p = 0.052). At the time of diagnosis of metastatic complications, 12.5% of patients were receiving combination antifungal therapy including amphotericin B.

**Table 4:** Cause-specific hazard of all-cause death associated with metastatic infection in patients with *Candidozyma auris* candidemia, Spain, 2016–2024*

Model and variable	HR (95% CI)	p value
30 d		
Metastatic infection, unadjusted	2.01 (0.70–5.76)	0.196
Metastatic infection, adjusted	1.75 (0.58–5.29)	0.323
Age, per year	1.02 (0.99–1.04)	0.295
Charlson Comorbidity Index, per point	1.02 (0.91–1.14)	0.782
60 d		
Metastatic infection, unadjusted	2.45 (1.15–5.20)	0.020
Metastatic infection, adjusted	2.21 (1.04–4.67)	0.038
Age, per year	1.02 (0.99–1.04)	0.181
Charlson Comorbidity Index, per point	1.06 (0.98–1.14)	0.172
90 d		
Metastatic infection, unadjusted	2.38 (1.15–4.94)	0.020
Metastatic infection, adjusted	2.26 (1.09–4.67)	0.028
Age, per year	1.01 (0.99–1.03)	0.300
Charlson Comorbidity Index, per point	1.06 (0.97–1.14)	0.191

*Adjusted models included age (per year) and the Charlson Comorbidity Index (per point). Patients were followed until death or at the end of the corresponding follow-up horizon. HR, hazard ratio using cause-specific Cox proportional hazards models for all-cause death (n = 108).

### Susceptibility Profiles of Cases and Controls

Overall, echinocandins demonstrated MIC values within the susceptible range according to the tentative breakpoints proposed by the US Centers for Disease Control and Prevention. For anidulafungin, MIC_50_ (MIC at which 50% of isolates are inhibited) was 0.125 mg/L; MIC_90_ (MIC at which 90% of isolates are inhibited) was 0.25 mg/L. For caspofungin, MIC_50_ was 0.06 mg/L and MIC_90_ was 0.25 mg/L. For micafungin, MIC_50_ was 0.06 mg/L and MIC_90_ 0.125 mg/L. All isolates were susceptible to amphotericin B; MIC_50_ was 0.5 mg/L and MIC_90_ was 1 mg/L. In contrast, all isolates were resistant to fluconazole (MIC >256 mg/L). Voriconazole MICs were consistently >2 mg/L across all isolates. We observed no significant differences in MIC values between groups (Table 5).

**Table 5:** Antifungal susceptibility of *Candidozyma auris* isolates from patients with and without metastatic infection, Spain, 2016–2024*

Antifungal drug	p value	Metastatic infection absent			Metastatic infection present	
		MIC_50_, mg/L	MIC_90_, mg/L		MIC_50_, mg/L	MIC_90_, mg/L
Amphotericin B	0.521	0.5	1		0.5	1
Fluconazole	0.949	256	256		256	256
Itraconazole	0.440	0.25	0.5		0.125	0.5
Voriconazole	0.501	2	4		2	4
Caspofungin	0.416	0.06	0.125		0.06	0.25
Anidulafungin	0.192	0.125	0.25		0.125	0.25
Micafungin	0.853	0.06	0.125		0.06	0.125
Posaconazole	0.528	0.06	0.25		0.125	0.25
Isavuconazole	0.280	0.25	0.5		0.25	2

*MIC_50_, MIC inhibiting 50% of *C. auris* isolates; MIC_90_, MIC inhibiting 90% of *C. auris* isolates.

## Discussion

We identified metastatic complications in ≈25% of patients with *C. auris* candidemia; endocarditis was the predominant clinical manifestation. Those findings underscore the propensity of *C. auris* to cause invasive device-associated infections. The presence of intracardiac devices or prosthetic valves was strongly associated with metastatic dissemination in our primary analysis. In the sensitivity analysis restricted to patients who underwent echocardiography, the association remained similar in magnitude but was no longer statistically significant after adjustment. Although prolonged candidemia was associated with dissemination in bivariable analysis, it did not reach independent significance after accounting for competing risks. Crude mortality proportions did not differ significantly between groups, but time-dependent analyses identified an association between metastatic infection and increased mortality at 60 and 90 days. Metastatic infection was also associated with prolonged antifungal treatment; hospital stay was longer among patients with metastatic infection, but the difference was not statistically significant. We observed no clear differences between groups with respect to initial antifungal strategies or antifungal susceptibility profiles, including MIC patterns.

Few published studies have described the incidence of metastatic complications after *C. auris* candidemia. In a multicenter study (*20*), metastatic infection was identified in 13 (8.02%) of 162 patients; endocarditis was the most common complication, consistent with our findings. Another study (*19*) reported metastatic infection in 10 (27.02%) of 37 cases, a proportion comparable to that observed in our cohort; however, neither the spectrum of complications nor associated outcomes were specified. Lower rates of metastatic infection were previously described (*18**,**22*); as in our cohort, a substantial proportion of patients in those studies received combination antifungal therapy. Beyond those cohorts, published experience is confined to isolated case reports and small series, dominated by endocarditis in recipients of left ventricular assist devices (*15*,*17*) and by central nervous system infection associated with external ventricular drains or intracranial pressure monitors (*14*,*21*,*23*); osteoarticular involvement is described only occasionally (*13*,*16*). That device-associated pattern mirrors the one observed in our cohort, although it may also reflect publication bias toward severe or device-related manifestations. Interpretation of the available literature is further limited by considerable heterogeneity in study design, diagnostic strategies, and outcome reporting, which hinders reliable comparisons and underscores the value of cohort-based analyses such as our investigation.

The incidence of metastatic infection in our study (22.2%) is situated within the broad range previously reported for metastatic complications in candidemia, estimated at 4%–50% (*4*–*7*,*26*). Our findings are consistent with higher rates described in critically ill and younger patient populations. The CandidICU multicenter study in France identified >1 secondary focus of infection in 21.8% of screened ICU patients with candidemia (*26*), whereas a separate study in children and young adults reported disseminated disease in 25% of evaluated episodes (*6*). A notable finding of our study is the high incidence of endocarditis (7.4%), which exceeded that reported in other previously published cohorts; that difference could be attributable to the greater prevalence of intracardiac devices and prosthetic valves in our population and the strong association of cardiac devices with metastatic infection in our cohort. Such an association is biologically plausible, given the well-recognized ability of *C. auris* to form biofilms on abiotic surfaces, which enables persistence and infection of intracardiac devices (*27*). Prosthetic heart valves were the most frequent type of cardiac foreign material in both groups, whereas left ventricular assist devices were observed only among patients with metastatic infection. However, the small number of patients within each device subtype precludes reliable subtype-specific inference. Similarly, one study (*7*) reported an overall endocarditis incidence of 0.5%, increasing to 3.6% among patients who underwent echocardiography evaluation; of note, 1 of 4 patients with endocarditis had a prosthetic valve. Another study (*28*) reported overall endocarditis incidence of 2.5% among patients with candidemia and identified preexisting valvular heart disease as an independent risk factor, further supporting the contribution of structural cardiac abnormalities to the development of *Candida* endocarditis. In addition, several case series have described *C. auris* candidemia in patients with left ventricular assist devices, frequently documenting valvular or device-associated vegetations, consistent with the pattern observed in our cohort (*15*,*17*). Those observations are further reinforced by data from our center in transplant populations, where a particularly high risk for *C. auris* infection has been identified among cardiac transplant recipients (*29*).

In our cohort, ocular involvement occurred in 0.9% of patients, a substantially lower proportion than that reported in studies of *Candida* spp. candidemia that identified ocular complications in 10%–16% of systematically screened patients (*7*,*30*,*31*). That finding also differs from one in which panophthalmitis was the most frequently reported complication after *C. auris* candidemia (*18*).

Although candidemia duration was associated with metastatic dissemination in bivariable analysis (p = 0.034), it was not confirmed as an independent predictor in the multivariable Fine–Gray model. A landmark analysis restricted to day-5 survivors, performed to avoid immortal time bias inherent to the definition of persistence, showed a higher but not statistically significant risk for metastatic dissemination with persistent candidemia (sHR 2.67 [95% CI 0.86–8.32]; p = 0.091), consistent with previous reports in young adults (*6*) and in ICU settings (*26*) but likely underpowered given the sample size.

In crude comparisons, the risk for death did not differ between patients with and without metastatic infection and was lower among those with metastatic infection at day 30. The apparent paradox reflects the fact that patients must survive long enough for dissemination to be diagnosed. When we modeled infection as a time-dependent exposure, the direction of the association reversed; we observed that cause-specific hazard of death was almost twice as high at 60 and 90 days than at day 30. Our findings are therefore consistent with those published previously (*20*) that identified disseminated disease after *C. auris* candidemia as an independent predictor of death. Patients with metastatic disease in our cohort also required significantly longer antifungal therapy (43 vs. 23 days) and longer hospitalization, although the difference was not statistically significant (47 vs. 22.5 days), consistent with previous studies showing that complicated candidemia frequently requires prolonged treatment courses and additional therapeutic interventions (*7*,*26*).

Our study cohort provided detailed clinical and microbiological information on the range of metastatic complications after *C. auris* candidemia in a hospital where the pathogen has remained endemic since its emergence in 2016. The relatively large size of the cohort and the extended period of data collection enabled us to estimate the frequency of metastatic involvement and identify factors associated with its development. In contrast to previous reports, which have largely been limited to case reports or small case series (*13*–*17*,*21*,*23*), our study provided cohort-based estimates of metastatic dissemination.

The retrospective, single-center design might limit the generalizability of our findings, particularly across settings with differing patient populations, diagnostic approaches, or antifungal stewardship strategies. Furthermore, metastatic involvement might have been underestimated; blood cultures have limited sensitivity, and advanced imaging, or invasive diagnostic procedures were not performed systematically. Echocardiography was performed more frequently in patients with metastatic infection, raising the possibility of diagnostic ascertainment bias. Although the association between cardiac devices and metastatic infection remained similar in magnitude in the sensitivity analysis restricted to patients who underwent echocardiography, it was no longer statistically significant after adjustment. Therefore, residual detection bias cannot be excluded. Finally, the relatively small number of patients with metastatic infection limited statistical power and the precision of the effect estimates, particularly in the multivariable and landmark analyses. We recommend caution in interpreting the associations we observed; they should not be considered definitive evidence of causality.

Our findings expand the body of knowledge of *C. auris* candidemia, showing that metastatic complications are not uncommon and most frequently involve the cardiovascular system, especially the heart. The association with intracardiac devices and the trend toward higher risk with persistent candidemia observed in the landmark analysis suggest that dissemination may be driven by both device-related and fungemia-related factors. Those results underscore the need for heightened clinical vigilance and targeted cardiac assessment in at-risk patients.

Although it remains uncertain whether metastatic complications occur more frequently in *C. auris* candidemia than in infections caused by other yeast species, the proportion observed in our cohort was notable. That finding supports the potential value of systematic colonization screening in healthcare settings where *C. auris* has been identified, particularly because previous studies suggest that colonization is associated with an increased risk for subsequent invasive infection (*32*).

Future studies should compare the risk for and spectrum of metastatic complications of *C. auris* candidemia with those observed in other yeast infections. Such comparative analyses are essential to determine whether *C. auris* candidemia itself constitutes an independent risk factor for dissemination, beyond host-related and device-related predictors. Multicenter prospective studies with systematic screening for deep-seated foci will establish the relative contributions of pathogen-specific versus host-related factors in the pathogenesis of *C. auris* dissemination.

As of September 2026, international guidelines recommend fundoscopy after candidemia in selected patients, particularly those experiencing ocular symptoms, persistent fungemia, immunosuppression, or an inability to report visual disturbances. Similarly, echocardiography is advised in patients with suspected endocarditis (e.g., new cardiac murmur or embolic phenomena), persistent candidemia, underlying valvular disease, or the presence of cardiac implantable devices (*33*–*35*), although further research is needed to determine the optimal timing for echocardiographic assessment after candidemia diagnosis. Our findings support this risk-based approach and highlight the importance of echocardiographic evaluation, particularly in patients with prosthetic valves or intracardiac devices; those subgroups exhibited the highest risk for metastatic complications. 
